# Propane Dehydrogenation Catalyzed by ZSM-5 Zeolites. A Mechanistic Study Based on the Selective Energy Transfer (SET) Theory

**DOI:** 10.3390/molecules20022529

**Published:** 2015-02-02

**Authors:** Ragnar Larsson

**Affiliations:** Chemical Engineering II, University of Lund, P.O. Box 124, SE-22100 Lund, Sweden; E-Mail: Ragnar.Larsson@chemeng.lth.se; Tel.: +46-46-121-123

**Keywords:** propane dehydrogenation, zeolite catalysis, SET, selective energy transfer, activation energies, molecular vibrations, resonance, vibrational quantum numbers, isokinetic temperature

## Abstract

Experimentally determined activation energies of propane dehydrogenation catalyzed by ZSM-5 zeolites have been used to test the SET theory. The basis of this theory is that the catalyst system transfers vibrational energy via a resonance process to a specific vibration mode of the reacting molecule. Being excited up to a certain number of vibrational quanta the molecule is brought to reaction. By analyzing the above-mentioned activation energies we found the wave number of this “specific mode” to be 1065 cm^−1^. This is very close to the rocking vibration of propane (1053 cm^−1^). We suggest that the propane molecule reacts when excited so that the CH_3_ group has been forced towards a flat structure with a carbon atom hybridization that is more sp^2^ than sp^3^. Consequently there is no way for three H-atoms to bind to the carbon and one of them must leave. This is the starting point of the reaction. The isokinetic temperature of the system was found as T_iso_ = 727 ± 4 K. From the SET formula for T_iso_ when both energy-donating (ω) and energy-accepting (ν) vibrations have the same frequency, *viz.*, T_iso_ = Nhcν/2R, we obtain ν = ω = 1011 ± 6 cm^−1^. This agrees rather well with the CH_3_ rocking mode (1053 cm^−1^) and also with asymmetric “TO_4_” stretching vibrations of the zeolite structure (ω).

## 1. Introduction

The dehydrogenation of propane over zeolite catalysts is of great industrial importance as the product, propene, is used for further synthesis. Many attempts have been made to explain the reaction mechanism from *ab initio* calculations [1,2] The measurement of the temperature dependence of the reaction rate over ZSM-5 zeolites [3], however, makes it possible to directly determine the nature of the reacting species from the model of Selective Energy Transfer (SET), developed by the present author [4,5]. The basic idea of this model of catalytic reactions is that energy is transferred via a resonance effect from a vibration within the catalyst system (ω) to one specific vibration of the reacting molecule (ν). The rate of such an energy transfer is treated in classical physics and we put the rate of the chemical reaction equal to the sum of such transfers from one energy level to the next, up to a number of vibrational levels, so that the molecule is so excited that it reacts. The method is described in detail in a recent review [5], but we can state here that the more vibrational quanta needed to excite the molecule to such a structural form that it fits into the structure of the catalyst, the larger is the activation energy. Thus, the nature of the catalyst determines the size of the activation energy.

The main significance of SET is that it makes it possible to understand the mechanism of the reaction. Such an analysis is found in the present paper. It is also important that the fundamental ideas of SET leads to a linear relation between the pre-exponential factor A and the activation energy Ea, in the Arrhenius expression for ln k, *i.e.*, the so called Compensation effect. An application of SET might be to predict a certain substance’s ability as catalyst for a specific reaction. Such a use of SET has, however, not been done yet.

## 2. Results

In Table 1 we have reproduced the data on activation energies for a series of ZSM-5 catalysts determined by Yun and Lobo [3]. The activation enthalpy was calculated as ∆H^#^ = Ea − RT, where T is the mean temperature of the experiments. The following treatment rests on the observation [6,7] that there is a stepwise change in the activation energies (or activation enthalpies, ∆H^#^) for the same reaction and slightly different catalysts.

**Table 1:** Experimental data and subsequent treatment. ∆H^#^ = Ea − RT, where T is a mean value (770 K) for the temperature range of the measurements. ∆(∆H^#^) = abs $(∆H_{i}^{\#}-H_{i+1}^{\#}).$ n* means the number of a certain common term that seems to appear in such a difference. A vibrational quantum number is estimated as described in the text.

Catalyst	Ea kJ/mol	∆H^#^ kJ/mol	∆(∆H^#^) kJ/mol	n*	∆H^#^/13.1	n
H[Fe]ZSM-5	115	108.6	58	4	8.290	8
Na[Fe]ZSM-5	77	70.6	38	3	5.389	5
Na[Fe]ZSM-5 ^a^	104	97.6	27	2	7.450	7
[Fe]ZSM-5 ^b^	98	91.6	6		6.992	7
[Fe]ZSM-5 ^c^	99	92.6	1		7.069	7
H[Al]ZSM-5	173	166.6	74	6	12.718	13
Sum			197	15		

a, exchanged after reaction; b, steamed; c, Na—steamed; E_0_ = 197/15 = 13.1 kJ/mol.

Hence we form the consecutive differences between the reported enthalpies of activation. This is done to try to find the least common term that makes up the data under consideration.

This is admittedly a somewhat subjective process, but to exemplify, we can guess that there might be—in the data of Table 1—such a term of the order of about 13 as 3 × 13 = 39 and 2 × 13 = 26; *cf*. Column ∆(∆H^#^), 38 and 27.

The next step of calculation is therefore to estimate the number of the least common term that appears in the various differences; neglecting such differences that are too small. *Cf*. Ref [5,7]. We denote this number as n* and calculate ∑n* as well as ∑∆(∆H^#^). This gives a reasonably accurate way to estimate the “least common term” that we call E_0_:

E_0_ = ∑∆(∆H^#^)/∑n* (1)

Now, if the term E_0_ constitutes the differences between activation enthalpies, then it should also build up the activation enthalpies themselves. Thus:

∆H^#^ = n E_0_ (2)

where n is the vibrational quantum number up to which the reacting molecule has to be excited for reaction to occur.

**Figure 1:**
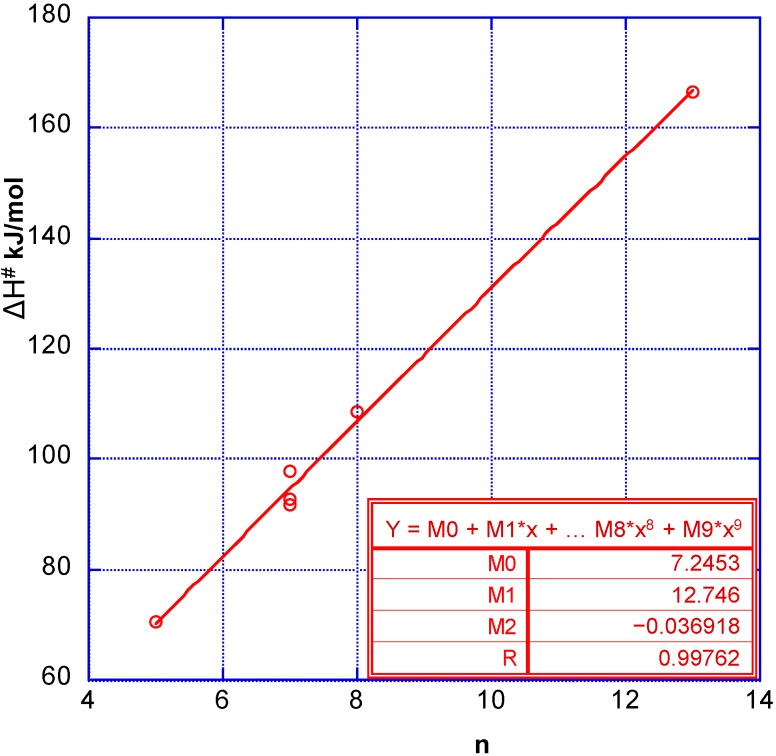
∆H^#^ plotted as a function of the corresponding vibrational quantum number, n. The six points from Table 1 were fitted to a second order function. The result is given within the graph.

By using Equation (2) we can get a rough estimate of these quantum numbers (n) for each of the reacting systems. The thus obtained couples of ∆H^#^ and n are plotted in a graph (Figure 1) and fitted to a second order relation [7]:

∆H^#^ = M0 + M1 n +M2 n^2^ (3)

where M0 represents any influence from adsorption of the reacting molecule at the solid catalyst, M1 gives the frequency (wave number) of the molecule in question. The second order term represents the anharmonicity of the vibration. Consequently M2 should be negative.

When treating the data of Figure 1 in the way described above, we obtain:

∆H^#^ = 7.25 + 12.75n − 0.0369n^2^ (4)

The magnitude of M0 is somewhat surprising, as normally (for metal particles or metal oxide particles as catalysts) one finds M0 to be very close to zero, indicating that the catalytic activation occurs in the free space outside the catalyst surface [5,8,9]. This is thus not so in the present case.

## 3. Discussion

From the reported data we can conclude that the wave number of the vibration that has to be exited, ν, is:

ν = 12.75 kJ/mol = 1065 cm^−1^ (5)

and the anharmonicity, conventionally designed as x_0_ can be estimated as:

x_0_ν = −0.0369 kJ/mol = −3.1 cm^−1^ (6)

One can now note that this value of ν is quite close to the rocking vibration of the CH_3_ group of the propane molecule, *viz*. ν_20_(b1) = 1053 cm^−1^, following the tabulation and notation of Herzberg [10]. The relatively low value of the anharmonicity also indicates a bending motion.

Now, what does this imply? In a mechanistic interpretation, we see that the more the molecule is forced into this specific vibration, the more the tetrahedral sp^3^ hybridization of the carbon atom turns into an sp^2^ planar state. Consequently, the third hydrogen atom is less and less firmly bound to the carbon atom and can easily be intercepted by, e.g., an oxygen atom of the zeolite structure.

At the same time the middle carbon atom reaches a state of sp^2^ hybridization as it is interacting with the first carbon atom forming a “double-bond”. This means that also for this carbon atom, one of the hydrogen atoms becomes more and more loosely bound and can—also—be intercepted by the zeolite structure.

The size of the vibration leading to reaction (1065 cm^−1^) suggests that its corresponding vibration of the catalyst system (ω) would be an asymmetrical stretch of external or internal zeolite linkages [11]. In order to clarify this we have tried—in spite of the limited material—to estimate the isokinetic temperature, T_iso_.

**Figure 2:**
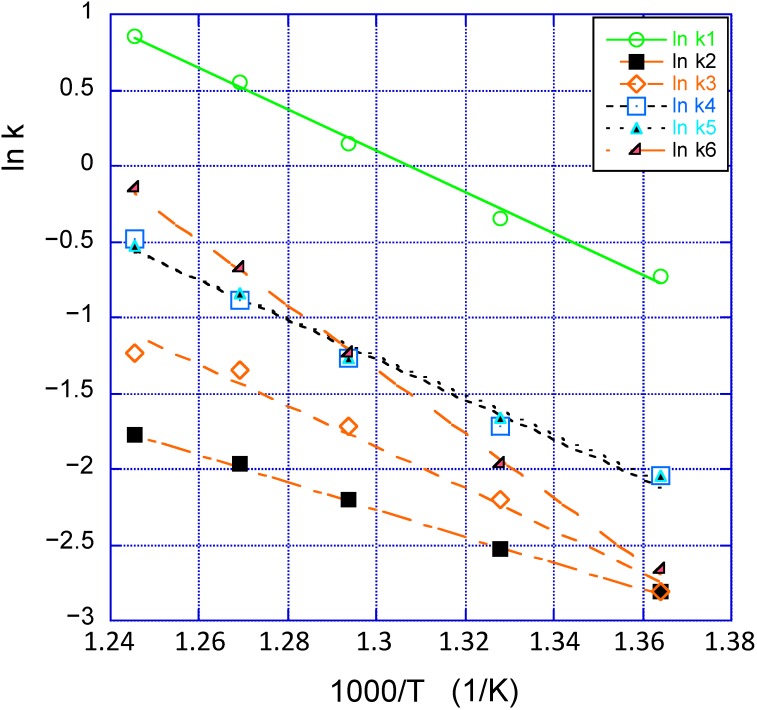
Arrhenius plots for the available data [3].

Figure 2 illustrates the Arrhenius relation between ln k and 1/T. Here ln k was derived from the A and Ea data of Yun and Lobo [3]. The values are given in the head of Figure 2. One notes that three of these lines (red-colored) cut in one and the same point, with a mean value of 1000/T = 1.376 ± 0.005 K^−1^ corresponding to T_iso_ = 727 ± 4 K (had a few more catalysts been included in the investigation, one might very well have found a “mate” to the upper line that most probably would have cut the other one at a similar temperature. This is not uncommon [12,13,14].

The relation between ln k and 1/T is described by Equation (1a) of [5]: as

ln k = ln Z + ω(ν^2^ − ω^2^)^−1^{±π/2 − arctg[0.5νω(ν^2^ − ω^2^)^−1^]}h c N∑ν_i_ − Ea/RT (7)

where ν and ω means the wave numbers of the critical vibration of the reacting species and that of the energy donating catalyst. Z means the part of the pre-exponential term that is independent of vibrational influence.

But:

h c N ∑ν = Ea
(8)

and hence, when Ea = 0:

ln k_iso_ = ln Z (9)

So, any variation of the crossing point of the Arrhenius lines must depend on variations in the entity Z. Perhaps of morphological nature, perhaps composition of the catalyst or reactant (in Ref. [14], where a difference of ∆ln k_iso_ = 0.75 was found between two groups of reactions, with seven and five members, respectively, we concluded that the difference originated from positions of substituents in the molecules that reacted—in one case *ortho*-positions, in the other *meta*- or *para*-positions). Incidentally, one notes that the combination of Equations (7) and (8) gives the so called Compensation effect.

The numbering of the rate constants follows the order of data in Table 1.

Previously [4] it has been deduced that the isokinetic temperature can be described as a function of ν and ω:

T_iso_ = NhcR^−1^ (ν^2^ − ω^2^) ω^−1^{±π/2 − arctg[0.5νω(ν^2^ − ω^2^)^−1^]}^−1^ (10)

However, for the condition that ν = ω, a much more simple relation holds [4]:

T_iso_ = Nhcν/2R = 0.719ν (11)

From the relation (11) and the value for T_iso_ found above, we can conclude that ν = ω = 1011 ± 6 cm^−1^. This is in good agreement with what we have found above (1065 cm^−1^), the data for the propane rocking vibration (1053 cm^−1^) [10], and also with what might be expected for an asymmetric “TO_4_” stretching vibration of a zeolite structure [11]. Such a TO_4_ structure has a high IR absorption intensity and thus also a high IR emission intensity.

## 4. Conclusions

From the above discussion we can conclude that zeolites are good catalysts—at least for dehydrogenation of alkanes—because their structures are built of strongly polar bonds and consequently they emit strong infrared radiation. This radiation couples via resonance to a suitable vibration mode of the reacting molecule. In the present case we find that this vibration mode is the CH_3_—rocking mode of propane. The sp^3^ hybridization of the end—carbon is forced towards an sp^2^ hybridization. In this way one hydrogen is expelled and furthermore, the route towards a C-C double bond is opened, requiring an sp^2^ hybridization also at the middle carbon atom. This, in its turn, results in the expulsion of a hydrogen atom also from the middle carbon atom.

The combination of these two atoms to a complete hydrogen molecule, H_2_, depends on the availability of a suitable H atom landing site. If this site is easily available, the extraxtion of the H-atom does not require any strong C-C bending from the rocking mode, and consequently the activation energy is not high. If, on the other hand, the landing site is so situated that the extraction of an H-atom from propane is difficult, more vibrational quantum states must be engaged and the activation energy is considerable. It is in this way, that the structure of the zeolite determines the size of the activation energy.
